# Poly (ADP-Ribose) and α–synuclein extracellular vesicles in patients with Parkinson disease: A possible biomarker of disease severity

**DOI:** 10.1371/journal.pone.0264446

**Published:** 2022-04-08

**Authors:** Fabrice Lucien, Eduardo E. Benarroch, Aidan Mullan, Farwa Ali, Bradley F. Boeve, Michelle M. Mielke, Ronald C. Petersen, Yohan Kim, Cole Stang, Emanuele Camerucci, Owen A. Ross, Zbigniew K. Wszolek, David Knopman, James Bower, Wolfgang Singer, Rodolfo Savica

**Affiliations:** 1 Department of Urology, Mayo Clinic, Rochester, Minnesota, United States of America; 2 Department of Neurology, Mayo Clinic, Rochester, Minnesota, United States of America; 3 Department of Health Science Research, Mayo Clinic, Rochester, Minnesota, United States of America; 4 Department of Neuroscience, Mayo Clinic, Jacksonville, Florida, United States of America; 5 Department of Clinical Genomics, Mayo Clinic, Jacksonville, Florida, United States of America; 6 Department of Neurology, Mayo Clinic, Jacksonville, Florida, United States of America; Johns Hopkins, UNITED STATES

## Abstract

**Background/Objective:**

Despite multiple attempts, no surrogate biomarker of Parkinson disease (PD) has been definitively identified. Alternatively, identifying a non-invasive biomarker is crucial to understanding the natural history, severity, and progression of PD and to guide future therapeutic trials. Recent work highlighted alpha synuclein-containing extracellular vesicles and Poly (ADP-ribose) polymerase (PARP-1) activity as drivers of PD pathogenesis and putative PD biomarkers. This exploratory study evaluated the role of alpha-synuclein-positive extracellular vesicles and PARP-1 activity in the plasma of PD patients as non-invasive markers of the disease’s severity and progression.

**Methods:**

We collected plasma of 57 PD patients (discovery cohort 20, replication cohort 37) and compared it with 20 unaffected individuals, 20 individuals with clinically diagnosed Alzheimer’s disease, and 20 individuals with dementia with Lewy bodies. We analyzed alpha-synuclein-positive extracellular vesicles from platelet-free plasma by nanoscale flow cytometry and blood concentrations of poly ADP-ribose using sandwich ELISA kits.

**Results:**

Median concentration of α-synuclein extracellular vesicles was significantly higher in PD patients compared to the other groups (Kruskal-Wallis, p < .0001). In the discovery cohort, patients with higher α-synuclein extracellular vesicles had a higher Unified Parkinson Disease Rating Scale score (UPDRS III median = 22 vs. 5, p = 0.045). Seven out of 20 patients (35%) showed detectable PAR levels, with positive patients showing significantly higher levels of α-synuclein extracellular vesicles. In the replication cohort, we did not observe a significant difference in the PAR-positive cases in relationship with UPDRS III.

**Conclusions:**

Non-invasive determination of α-synuclein-positive extracellular vesicles may provide a potential non-invasive marker of PD disease severity, and longitudinal studies are needed to evaluate the role of α-synuclein-positive extracellular vesicles as a marker of disease progression.

## Introduction

Parkinson disease (PD) and dementia with Lewy Bodies (DLB) are common disorders related to the accumulation of α-synuclein aggregates as Lewy bodies and Lewy neurites in the nervous system [1, 2]. Lewy bodies correlate with intracellular toxicity and cell-death-promoting events, including oxidative stress, impaired vesicular transport, lysosomal function, and neuroinflammation. Spatial progression of Lewy neuropathology may reflect spreading of abnormal fibrillary α-synucleins between cells [3–5]; however, the underlying propagation mechanisms are unclear. One possible spreading mechanism may be via extracellular vesicles (EVs), including exosomes [6]. Alpha-synuclein is a ubiquitous protein presenting on EVs [7]. Whether α-synuclein positive EVs are relevant in disease progression is undetermined [8].

In addition to impaired vesicular dynamics promoting cell-to-cell spreading, oxidative stress, mitochondrial dysfunction, neuroinflammation, and other interactive mechanisms may drive the pathological cascade leading to PD and DLB cell death. Poly (ADP-ribose) polymerase (PARP-1) mediates α-synuclein toxicity [9]. PARP-1, a fundamental component of DNA damage-response machinery, utilizes nicotinamide adenine nucleotide (NAD+) and ATP to synthesize poly-ADP-ribose (PAR) residues added to target proteins. Hyper-activated PARP-1 leads to regulated cell death or parthanatos. Parthanatos occurs in response to DNA damage, oxidative stress, inflammatory signals, and nitric oxide (NO). PARP-1 activation in PD may be secondary in part to excessive NO production [10] generated by LB accumulation [9]. PARP-1 activity can be elevated in the cerebrospinal fluid and brains of PD patients. In addition, in experimental mice models, PARP-1 induction increases cell death and leads to a faster, more diffuse spreading of the disease [9].

The function of α-synuclein-containing EVs in cell-to-cell transmission and excessive PARP-1 activation in neurotoxicity suggest that these biomarkers in peripheral PD blood/plasma dictate the pathodynamics of this disease. However, this has not been systematically explored. Our study sought to assess the role of α-synuclein-positive EVs and PAR levels in plasma as biomarkers of PD presence and progression and whether the combined determination of EVs (a putative surrogate of α-synuclein accumulation and impaired autophagy) and PAR (product of PARP-1 activity) better assesses PD pathobiology.

## Materials and methods

### Case identification/ascertainment

We used three tissue repositories to perform blinded experiments. We included 20 consecutive cases of PD from the Mayo Clinic Rochester Movement Disorders Division (1/1/2019-8/1/2019), all of which fulfilled the UK Brain Bank clinical diagnostic criteria for PD. A movement-disorders specialist examining patients confirmed criteria and diagnosis. We used two comparison cohorts. First, we included patients and controls from the biorepository of the Alzheimer Disease Research Center (ADRC) of Mayo Clinic, Rochester (collected from 2005–2018). Second, we included 20 unaffected individuals and 20 cases of clinically diagnosed AD dementia and DLB. All the cases and controls were age/sex matched. We used an additional 37 cases (replication cohort) with a diagnosis of PD from the Mayo Clinic Florida Clinical and Genetic Studies, all of which fulfilled the UK Brain Bank clinical diagnosis for PD.

### Approvals and consents

Mayo Clinic and Olmsted Medical Center Institutional Review Boards approved this study. Participating patients (or legally authorized representatives) provided informed written consent for the use of medical information for research.

### Detection of α-synuclein extracellular vesicles in plasma

Stored plasma samples were thawed and centrifuged at 13,000 x g for 5 minutes to remove aggregates. Plasmas were diluted 1/10^th^ in PBS, and 10 microliters of diluted plasma were incubated with fluorescent-labeled primary antibodies against total α-synuclein (syn211, Thermo Fisher) and CD235a (clone HI264, Biolegend). EV populations were quantified from platelet-free plasma by nanoscale-flow cytometry using the Apogee A60-Micro Plus (Apogee Flow Systems Inc.). Before sample analysis, A60-Micro Plus sensitivity for detecting EVs was evaluated using a reference bead mix, as previously described [11] (S1 Fig). Sample flow rate was set at 1.5μL/min for all measurements, and acquisition time held constant for all samples at 60 seconds. Event rate was kept below 5,000 events per second to avoid swarm effect. Antibody-matched isotypes in the same condition were used to subtract unspecific binding. Data were analyzed using FlowJo v10.5 software. A one-way ANOVA test and a nonparametric *t* test identified differences between the control and patient groups in terms of clinical variables.

### Western blot

We selected 500 microliters of plasma from a subset of PD patients and age-matched healthy donors. Plasma was subjected to ultracentrifugation 100,000 x g for 2 hours at 4C using a Beckman Coulter Optima XPN-100 ultracentrifuge with a SW 55 Ti rotor. Following lysis, 25 ug of total proteins were analyzed by SDS-PAGE and western blot. Antibodies were used as follows: CD63 (abcam ab231975, 1/1000), Hsp70 (SBI, EXOAB-Hsp70A-1, 1/1000), aSyn phosphoS129 (abcam ab51253, 1/1000), aSyn (abcam MJFR1, 1/2000). Goat HRP-anti-Rabbit (Sigma, A0545) was used at a final dilution of 1/10000. ECL Pico Plus and ChemiDoc XRS+ were used for chemiluminescence imaging of proteins.

### Measurement of poly (ADP-ribose) concentration in plasma

The sandwich ELISA kit (XDN-5114, Cell Biolabs Inc.), per manufacturer’s instructions, determined PAR plasma levels. Plasma samples (50 μl) and standards were tested in duplicate. Color change was measured using the Varioskan LUX (Thermo Fisher). Data were analyzed using GraphPad Prism 7.0.

## Results

### Demographics and clinical characteristics

We included 20 consecutive patients diagnosed with PD according to the UK Parkinson’s Disease Society Brain Bank diagnostic criteria [12]. All PD cases were examined in “ON” state. Median-onset age was 70.5 years; 30% (6/20) were women. Median Hoehn and Yahr Scale (HYS) was 1 (ranges: 1–3); median UPDRS part III was 15 (2–30). Tremor was present in 40% (8/20); 12\20 had significant rigidity; 6/20 had severe postural reflex impairment; 17/20 had bradykinesia; 9/20 (45%) had cognitive decline; and 4/20 had evidence of visual hallucinations. All patients reported a significant response to levodopa intake. Randomly selected ADRC participants included 20 age-matched, unaffected controls (CU), 20 with probable AD dementia, and 20 with DLB. The groups’ median ages were similar: CU, 74.6 years old; AD dementia, 74.6 years old; and DLB, 75.4 years old. In the replication cohort, median-onset age was 73 years; and 40% (15/37) were women. Median Hoehn and Yahr Scale (HYS) was 2 (Ranges: 1–3); median UPDRS part III was 16 (7–44).

### Elevation of circulating α-synuclein extracellular vesicles and red blood cell-derived EVs in Parkinson disease

**Fig 1:**
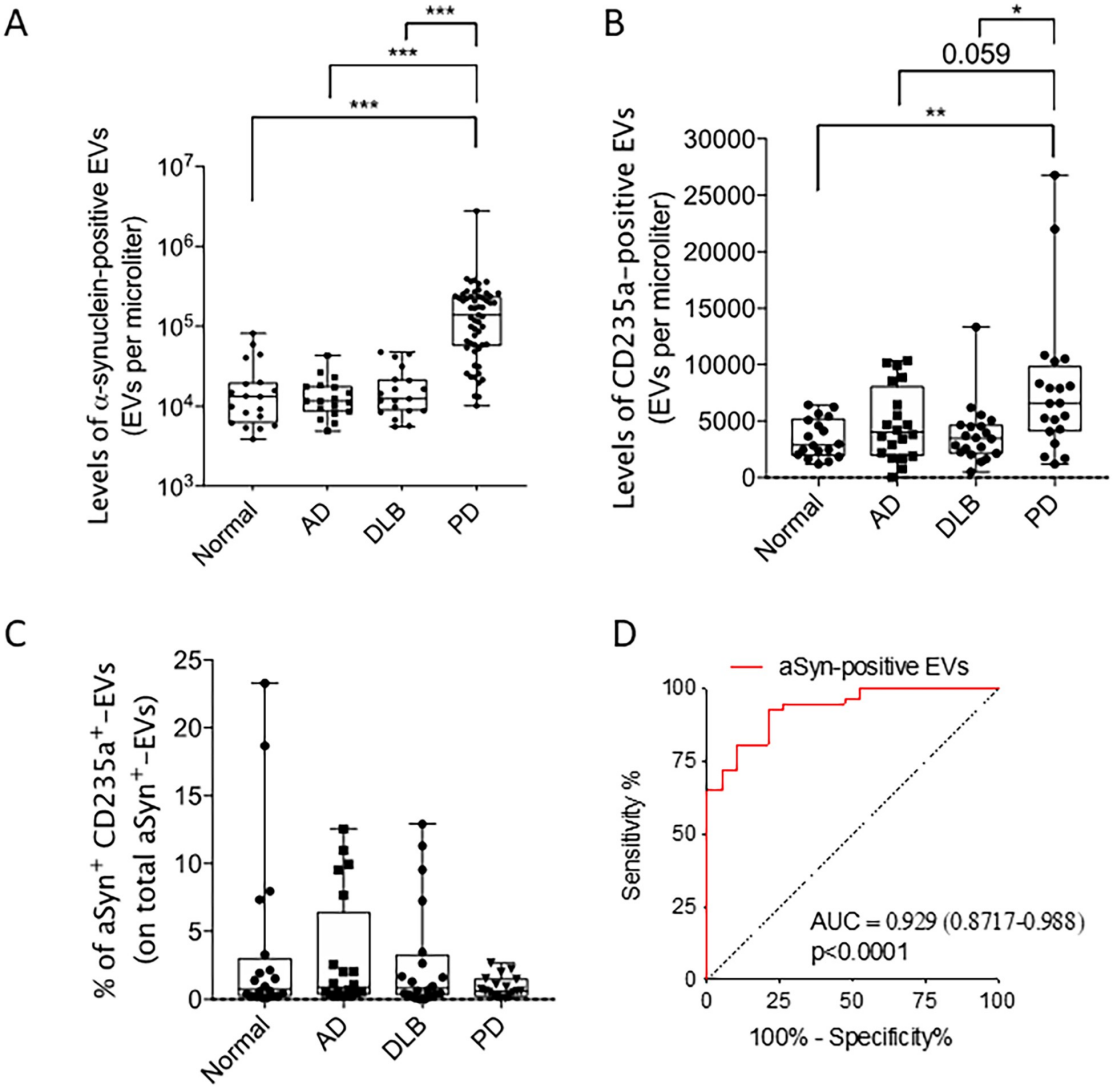
Levels of α-synuclein extracellular vesicles distinguish Parkinson disease (PD) patients from age-matched healthy individuals, Alzheimer disease (AD) patients, and patients having dementia with Lewy bodies (DLB). Comparison between levels of a-syn EVs in normal, AD, DLB, and PD patients. A) Levels of a-syn positive EVs in the four groups; B) Levels of CD32 a-syn EVs in the four groups C) Percentage of CD32 a-syn in the four groups; D) Receiving Operator Curve of identifying a-syn positive EVs compared to controls: sensitivity of 92.98% and a specificity of 78.95% at a cut-off of 20,700 α-synuclein-EVs per microliter of blood. *** indicates the first batch of samples where CD235a was used.

Median α-synuclein-EV concentration was significantly higher in PD patients than in other groups (138,8667 EVs/μl [10,173–2,758,267 min-max]) (Fig 1A). Median concentration of α-synuclein EVs was 13,253 Evs/μl (3,880–81,847 min-max) in normal individuals, 11,603 Evs/μl (4,920–43,207 min-max) in individuals with AD dementia, and 12,393 EVs/μl (5,553–47,800 min-max) in individuals with DLB. Prior work showed that red blood cells (RBC) are a major source of peripheral α-synuclein [25]. We sought to determine whether plasma-derived α-synuclein-EVs origin from RBC. We measured the concentrations of RBC-EV with the marker CD235a and determined the percentage of α-synuclein-EVs that were positive for CD235a (Fig 1B and 1C). Median RBC-EV concentration was significantly higher in the PD group (6,593 EVs/μl [1,200–26,827]) compared to the AD dementia and DLB groups (p = 0.059; Fig 1B). Median RBC-EV concentration was 2,910 EVs/μl (1,200–6,453 min-max) in the CU; and 4,017 EVs/μl (0–10,340) for individuals with AD dementia; and 3,480 EVs/μl (480–13,340) for individuals with DLB. The percentage of α-synuclein-EVs positive for CD235a (α-synuclein^+^CD235^+^EVs) did not differ significantly between groups (Fig 1C). The median percentage of α-synuclein^+^CD235a^+^EVs was 0.76%, 0.87%, 0.80%, and 0.604% in the CU, AD, DLB, and PD groups, respectively. We used a ROC curve (Fig 1D) to determine the diagnostic accuracy of α-synuclein-EV levels to distinguish PD patients from healthy individuals. The AUC was 0.929 (p<0.0001), with a sensitivity of 92.98% and a specificity of 78.95% at a cut-off of 20,700 α-synuclein-EVs per microliter of blood.

**Fig 2:**
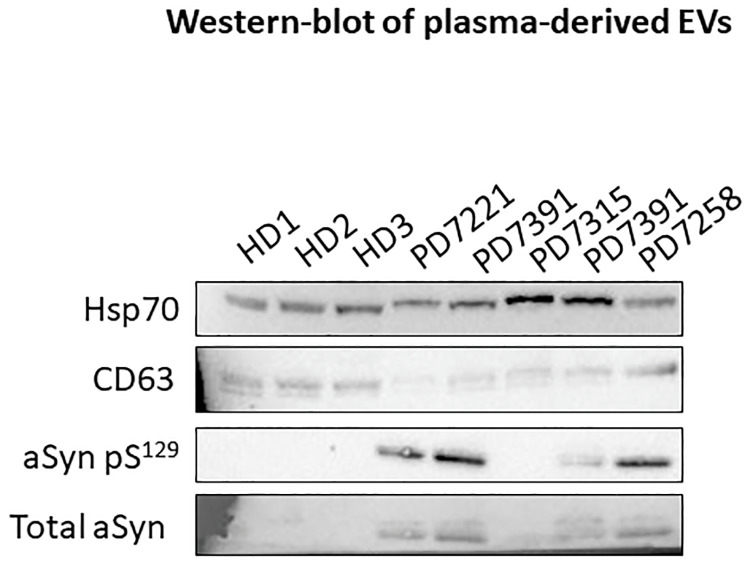
Western blot of plasma-derived EVs comparing healthy donors and PD patients. Total aSyn and aSyn S129 are detectable only on PD samples. No difference has been detected between total aSyn and aSyn S129.

To validate our findings, we analyzed the presence of phosphorylated and total α-synuclein in EVs isolated from blood of age-matched healthy individuals (N = 3) and PD patients (N = 5) (Fig 2). We used CD63 and HSP70 as positive control for EV-enriched proteins. Total α-synuclein and α-synuclein phosphoS129 were only detectable in 4 out of 5 samples of PD patients. No α-synuclein was detected in healthy donors. In addition, we did not observe any difference between total aSyn and aSyn S129 in PD patients, thus suggesting that both forms are found in peripheral EVs from PD patients.

Our observations suggest that peripheral α-synuclein EV do not origin from RBC and α-synuclein-EV levels can distinguish PD patients from healthy individuals free of cognitive impairment or parkinsonism and from patients with Alzheimer disease or Lewy body dementia.

### High UPDRS scores and elevated α-synuclein extracellular-vesicle levels

**Fig 3:**
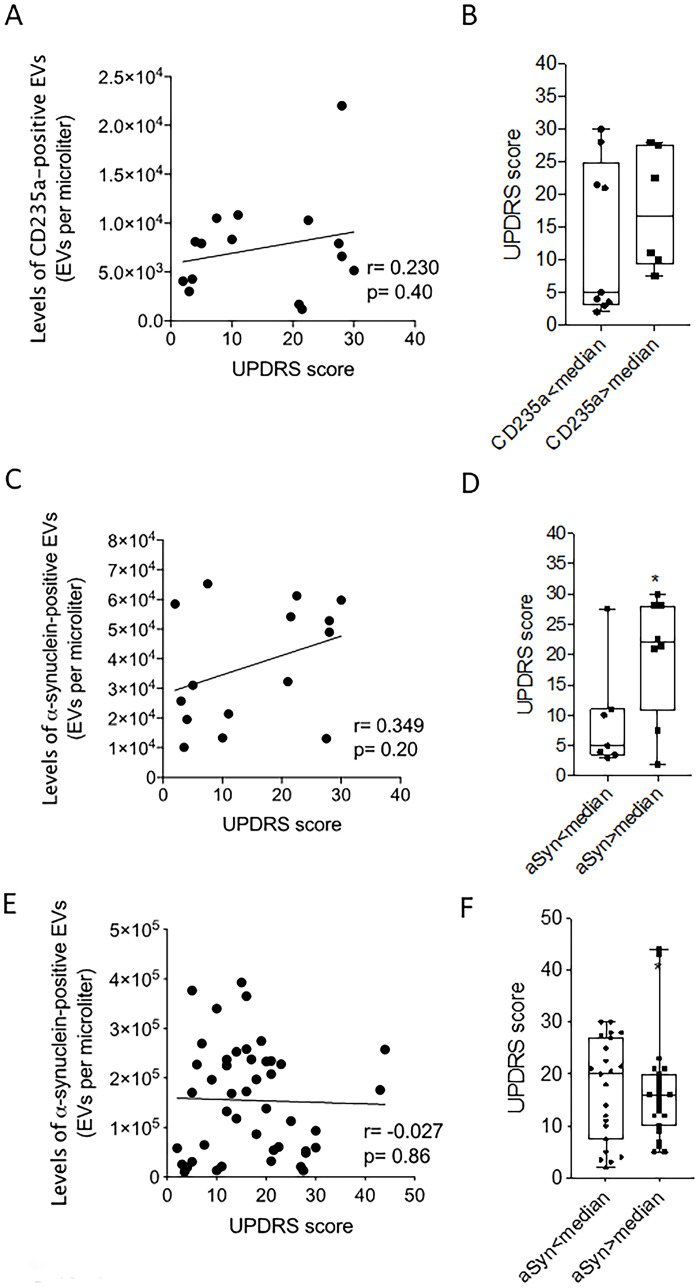
Levels of CD325a-syn EVs in PD and correlation with UPDRS scores. A) Levels of total a-syn EVs in PD and correlation with UPDRS scores; B) UPDRS scores and median levels of CD325a-syn EVs in PD; C) Levels of total a-syn EVs in PD and correlation with UPDRS scores; D) UPDRS scores and median levels of a-syn EVs in PD; E) Levels of total a-syn EVs in PD and correlation with UPDRS scores in replication cohort; F) UPDRS scores and median levels of a-syn EVs in PD in replication cohort.

Our analyses included UPDRS III as a disease-severity measure. In our original cohort, PD patients with available UPDRS scores (N = 15) had non-significant correlations between RBC-EVs and their UPDRS score (Fig 3A and 3B). We observed a moderate correlation between α-synuclein EV levels and UPDRS scores, which was insignificant (Pearson coefficient r = 0.349, p = 0.20) (Fig 3C). Interestingly, patients with higher α-synuclein EV levels showed higher UPDRS scores (median = 22 versus 5, p = 0.045) than patients with lower α-synuclein-median EV levels (Fig 3D). One patient out of 7 presented below-the-median levels of α-synuclein-EVs and a UPDRS score of 27.5. Inversely, 2 patients out of 8 presented high α-synuclein-EV levels and UPDRS scores of 2.0 and 7.5. The replication cohort revealed no correlation between α-synuclein-EVs and UPDRS scores (Pearson coefficient r = -0.027, p = 0.86) (Fig 3E and 3F). Similarly, we observed no significant difference when comparing UPDRS scores in patients above and below the median level of α-synuclein-EVs. These findings suggest that levels of α-synuclein-EVs at a single timepoint do not predict disease severity.

### Association of PAR plasma levels with aSyn-positive EVs and UPDRS scores in PD patients

**Fig 4:**
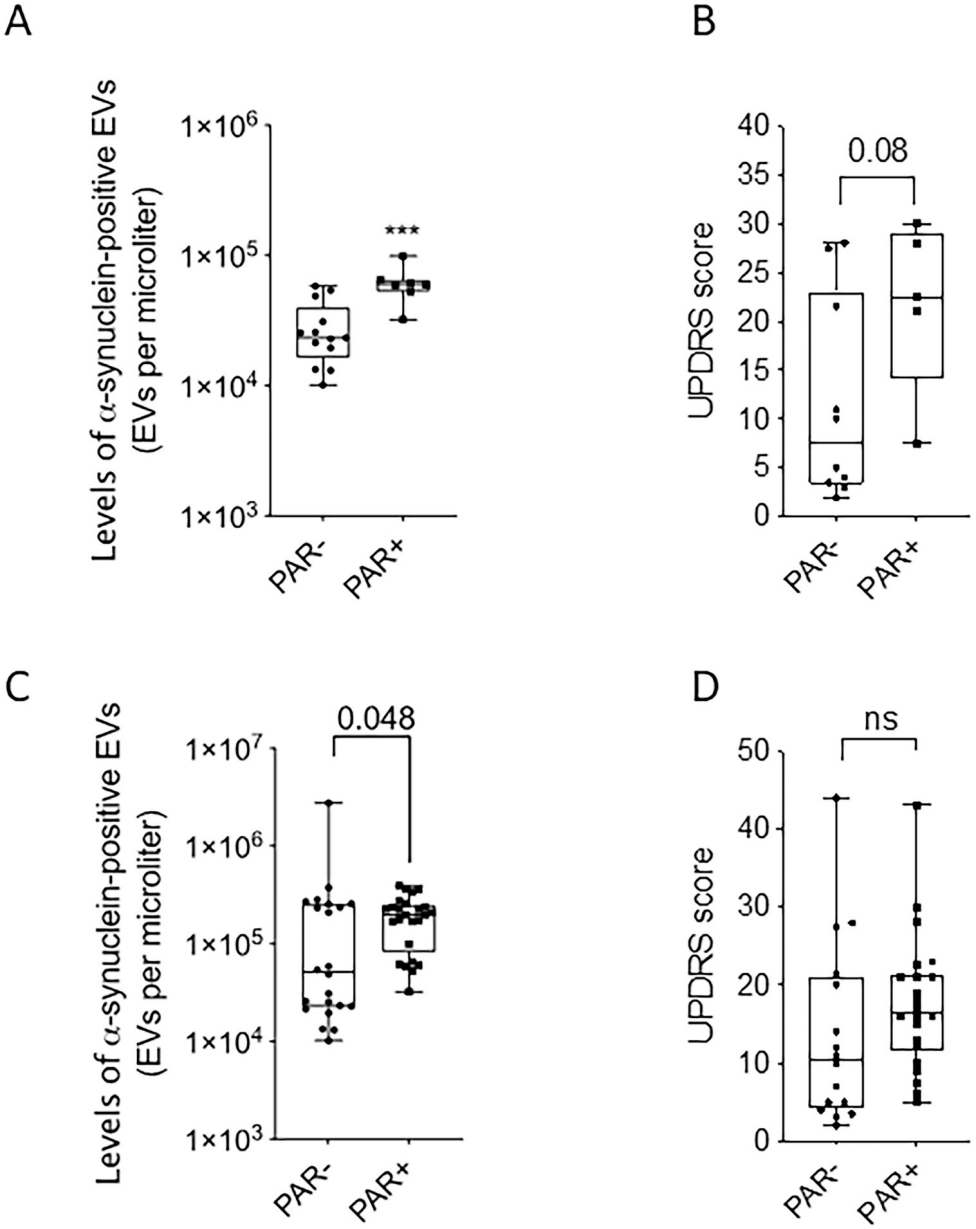
A) Levels of total a-syn EVs in PD and correlation with PAR activity in PD. B) UPDRS scores and PAR activity in PD; C) Levels of total a-syn EVs in PD and correlation with PAR activity in PD in replication cohort; D) UPDRS scores and PAR activity in PD in replication cohort.

We measured PAR plasma levels and determined the association with α-synuclein-EV levels and UPDRS scores (Fig 4). In the initial cohort, 7 out of 20 patients (35%) showed detectable PAR levels; and 13 out of 20 (65%) were undetectable. For patients with detectable PAR levels (PAR+), the median concentration was 255 pM (0.038–613 min-max). PAR+ patients had significantly higher α-synuclein-EV levels than PAR- patients (median 23,347 versus 59,800 EVs/μl–Fig 4A). Additionally, PAR+ patients had higher (albeit non-significant) UPDRS scores (median 22.5 versus 7.5; p = 0.08) (Fig 4B). Patients with detectable serum PAR levels and higher α-synuclein EV levels reported more severely progressive symptoms of disease (e.g., cognitive decline, hallucinations). In the second analysis, including the replication cohort, higher α-synuclein-EV levels were still found in PD patients with detectable PAR levels (p = 0.048) (Fig 4C). No significant difference was observed for PAR positivity and UPDRS scores (Fig 4D). A positive association between blood levels of α-synuclein-EVs and PAR positivity was observed, and the latter did not predict disease severity.

## Discussion

Diagnosis and prognostication for PD and related disorders still rest upon clinical criteria and clinical observation. Autopsy remains the gold standard for diagnosis despite increased knowledge of PD pathophysiology. Blood-based biomarkers are needed to predict disease severity and progression and, potentially, to identify specific subtypes of disease.

Recent ground-breaking studies have provided important molecular and biological insights on the role of α-synuclein in PD pathology. Transferring α-synuclein between neurons contributes to disease development and progression [13]. Active intercellular α-synuclein transfers occur through EV release [14]. EVs are submicron particles (50–1000 nm) released by cells carrying surface antigens and cargo from donor cells; thereby, they may serve as disease biomarkers in liquid biopsies [15]. In the context of synucleinopathies, EVs facilitate intercellular communication, contributing to LBD pathophysiology by transferring toxic forms of α-synuclein to healthy neurons. These findings suggest α-synuclein-positive EVs as a marker for diagnosis and progression of synucleinopathies [16]. Unlike previous reports using ELISA-based methods, our approach relies on direct enumeration of α-synuclein-positive EVs by nanoscale-flow cytometry, a state-of-the-art methodology for multi-parametric detection and antibody-based quantification of extracellular-vesicle subpopulations [11]. High-resolution flow cytometry to develop EV-based blood biopsies is attractive for clinical practice, as it requires no labor-intensive, time-consuming sample preparation. In addition, it requires a small sample volume (<20 μl), with readings available within 2 hours after collection.

Our study measured α-synuclein-positive EV levels in platelet-poor plasma of PD patients and determined differences between PD patients, age-matched CU individuals, and patients with other neurodegenerative diseases, including AD dementia and DLB. PD patients had higher concentrations of α-synuclein-positive EVs compared to other groups. These findings support previous studies demonstrating elevated α-synuclein-positive EV levels in the blood of PD patients compared to healthy controls [17–20].

Recently, two independent studies have suggested that α-synuclein-positive EVs discriminate between PD, DLB, and healthy controls with similar areas under the curve results [21]. Another study highlighted how phosphorylated versus non-phosphorylated α-synuclein-positive EVs can help in the diagnosis of PD [22], thus confirming our results. There will likely be a future role for α-synuclein-positive EVs in the confirmation of the clinical diagnosis of PD.

Surprisingly, we did not find any difference in levels of α-synuclein-positive EVs between DLB and healthy controls. Our findings are in line with two previous studies. Jiang et al. used a combination of L1CAM-derived EV immunocapture and ELISA for total α-synuclein [23]. No statistical difference was observed between DLB and healthy controls. In a second study, proteomic profiling of plasma-derived EVs isolated from DLB and healthy controls did not reveal alpha-synuclein as a biomarker of DLB [24].

Red blood cells have been shown to be the major source of α-synuclein in blood, representing >99% of total α-synuclein [25]. The remaining <1% in plasma may originate from other sources (e.g., neurons). To avoid contaminating RBC-derived α-synuclein, previous studies performed immuno-purification of brain-derived EVs using the L1CAM marker prior to quantifying α-synuclein levels [17]. We directly quantified α-synuclein levels on RBC-derived EVs and observed <1% of total α-synuclein detected on EVs originating from red blood. This suggests that, unlike RBC-derived EVs, α-synuclein is located on the EV outer membrane, and its extracellular accessibility is suitable for direct antibody-based detection [26].

We also assessed the relationship between α-synuclein-positive EV levels and poly ADP-ribose levels in the blood of PD patients. Interestingly, we found that PD patients with detectable blood PAR levels have significantly higher levels of α-synuclein-positive EV. Through a positive feedback loop, PARP-1 activity accelerates aSyn propagation and neuronal cell death [9]. Compared to healthy donors, cerebrospinal fluid of PD patients showed higher levels of poly(ADP)-ribose (PAR), a product generated from PARP-1 activity. While preliminary, our findings suggest that PARP-1/α-synuclein cross-talk involved in PD pathogenesis translates to systemic circulation of PAR and α-synuclein-positive EV levels.

Using the UPDRS score, we found no correlation between disease severity, PAR levels, and α-synuclein-positive EV levels. Future larger, longitudinal studies may elucidate this suggested relationship between PD severity and PARP. PD is a complex and multifactorial neurodegenerative disease, and in addition to the dominant role of alpha-synuclein, other mechanisms such as mitochondrial dysfunction, autoimmune response, and central and peripheral inflammation may be involved in the development and progression of PD [27–29]. In addition, several studies, including ours, have focused on exploring one blood component (immune cells, extracellular vesicles, cytokines, or soluble proteins) at a single timepoint. Considering the heterogeneity of the disease [8], it is unlikely that a single marker can capture the variability of disease through the population and within the different phases of the disease. Therefore, deep molecular profiling of longitudinally collected blood samples is critical for identifying multiple markers and capturing variability and endophenotypes of the disease.

Limitations of our study include its exploratory nature and the sample size. A larger longitudinal cohort study may confirm our findings and elucidate relationships between PARP activity and soluble α-synuclein release (e.g., aggregates, extracellular vesicles). In addition, we did not perform PARP activity in AD and DLB; in the future, these studies are needed to understand the relationship between PARP and neurodegeneration. Also, we were not able to collect multiple longitudinal samples from the same patients; therefore, we could not explore the correlation between the blood biomarkers and disease progression. We did not perform any experiment differentiation between the tetrameric, dimeric, and/or monomeric form of α-synuclein; in the future, our findings must be replicated to explore the possible difference between different toxic species of α-synuclein in PD. Finally, blood and CSF from matched patients must be tested in parallel to evaluate the performance of potential α-synuclein and PAR-based biomarker assays.

## Conclusions

In conclusion, our study suggests that non-invasive determination of α-synuclein-positive EVs, with or without PARP activity, offers a non-invasive marker of disease progression in PD and may also identify a subtype of PD with unique biological and biochemical characteristics. PARP inhibitors may represent a future adjuvant, neuroprotective approach to alleviate disease severity.

## Supporting information

S1 Fig Nanoscale flow cytometric detection of aSyn- and CD235a-positive extracellular vesicles in platelet-free plasma. A) Representative scatterplot and histogram of a mixture of nanosized beads (110 to 1,300 nm). B) Scatterplots of nanoscale flow cytometric detection of aSyn- and CD235a-positive EVs from platelet-free plasma. C) Antibody titration curves for alpha-synuclein and CD235a antibodies using platelet-free plasma (N = 3 technical replicates). Arrows indicate optimal concentrations for each antibody.
(DOCX)

S1 File (PDF)
