# Supplementary figures and images for: Poly (ADP-Ribose) and α–synuclein extracellular vesicles in patients with Parkinson disease: A possible biomarker of disease severity
Source: PLoS One. 2022 Apr 8;17(4):e0264446. doi: 10.1371/journal.pone.0264446 (PMC8993007; doi:10.1371/journal.pone.0264446)

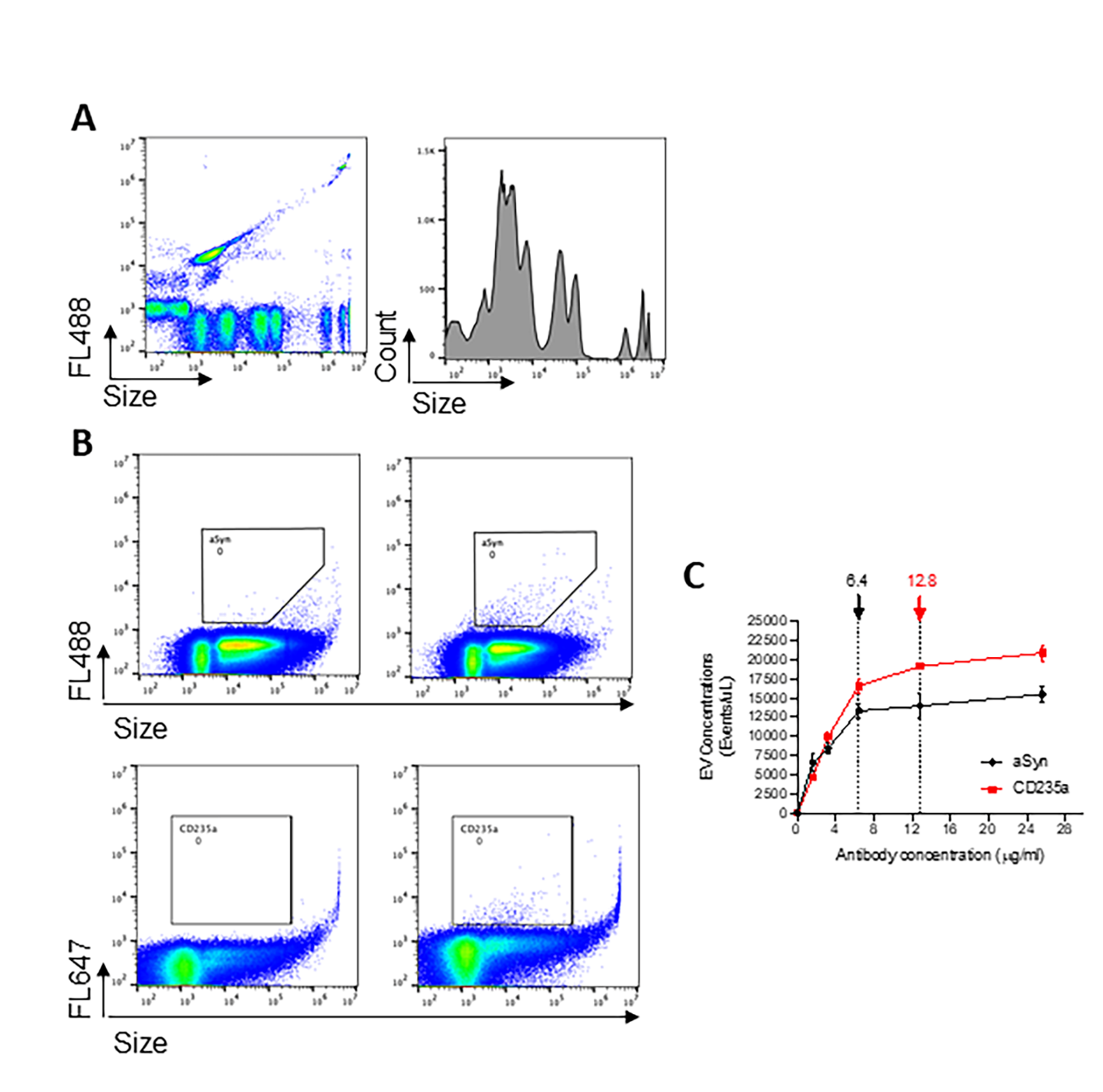

Supplement: S1 Fig — A) Representative scatterplot and histogram of a mixture of nanosized beads (110 to 1,300 nm). B) Scatterplots of nanoscale flow cytometric detection of aSyn- and CD235a-positive EVs from platelet-free plasma. C) Antibody titration curves for alpha-synuclein and CD235a antibodies using platelet-free plasma (N = 3 technical replicates). Arrows indicate optimal concentrations for each antibody. (DOCX) [file pone.0264446.s001.docx]

## Western-blot of plasma-derived EVs

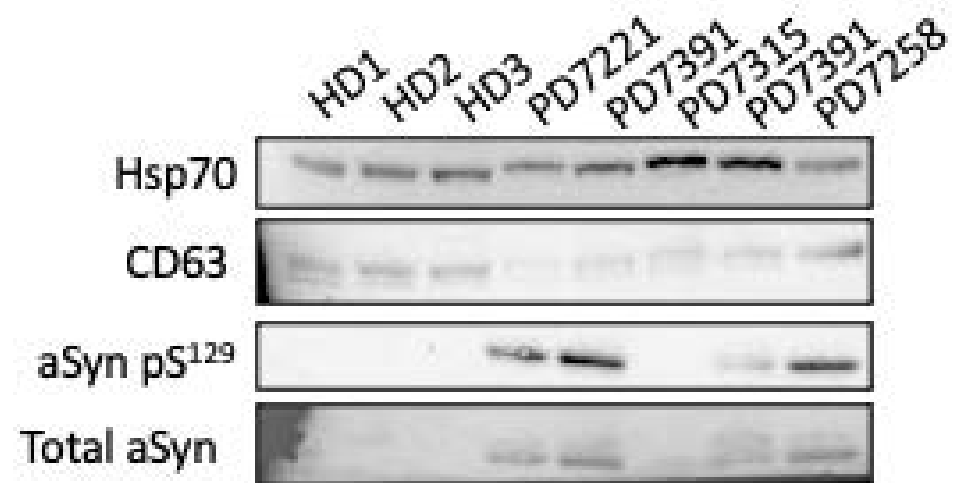

Hsp70

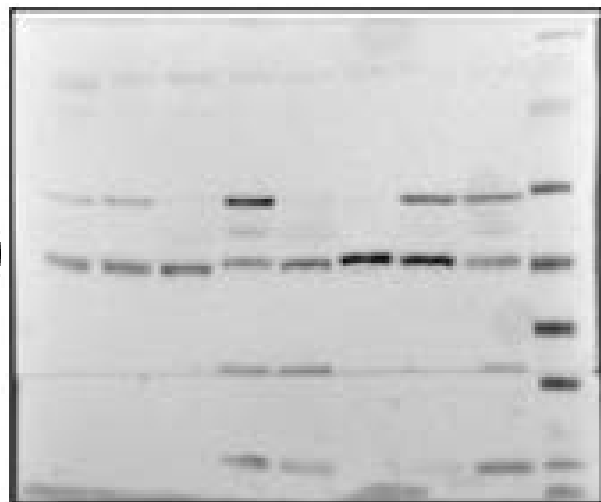

aSyn pS<sup>129</sup>

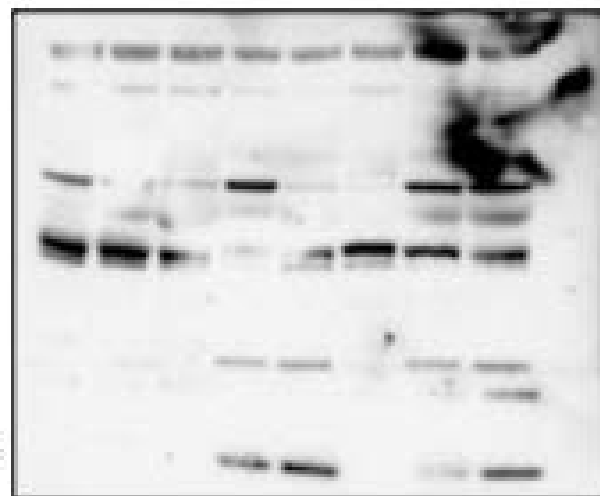

Total aSyn

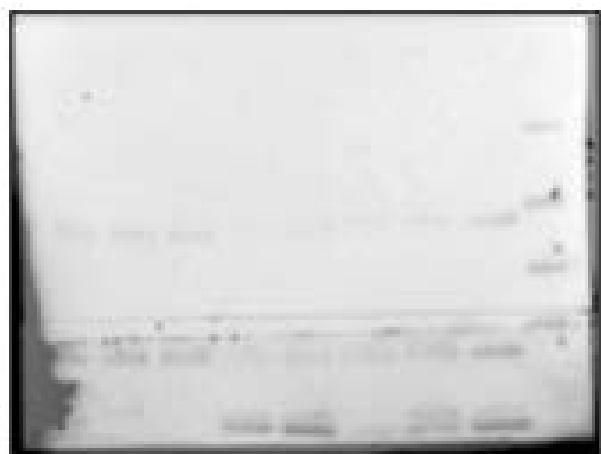

CD63

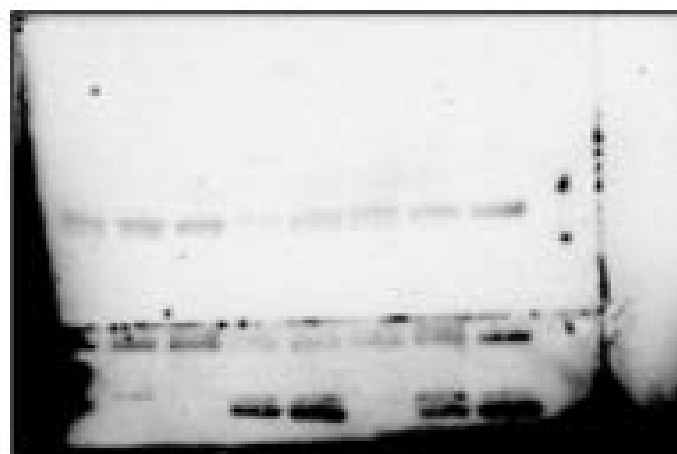

Supplement: S1 File — (PDF) [file pone.0264446.s002.pdf]
